# Diet-Induced Ceramide Remodeling as a Mechanistic Link to Cardiac Metabolic Dysfunction

**DOI:** 10.3390/nu18142239

**Published:** 2026-07-09

**Authors:** Manuela Giovanna Basilicata, Lucia Scisciola, Federico Capone, Elisabetta Trevellin, Pasquale Paolisso, Marta Belmonte, Ludovica Vittoria Marfella, Martina Zanzillo, Lorenzo Sabbatino, Luigi De Rosa, Nicola Celardo, Mario Acunto, Ada Pesapane, Rosaria Anna Fontanella, Nunzia Balzano, Nicoletta Lettera, Alberta Maria Maddalena Palazzo, Giovanni Tortorella, Rashmi Joshi, Asad Zia, Zeeshan Ulfat, Maryam Arshad, Paola Fioretto, Giuseppe Paolisso, Michelangela Barbieri

**Affiliations:** 1 Department of Advanced Medical and Surgical Sciences, University of Campania “Luigi Vanvitelli”, 80138 Naples, Italy; manuelagiovanna.basilicata@unicampania.it (M.G.B.); lucia.scisciola@unicampania.it (L.S.); ludovicavittoria.marfella@unicampania.it (L.V.M.); martina.zanzillo@studenti.unicampania.it (M.Z.); lorenzo.sabbatino1@unicampania.it (L.S.); luigi.derosa11@studenti.unicampania.it (L.D.R.); nicola.celardo@studenti.unicampania.it (N.C.); mario.acunto@studenti.unicampania.it (M.A.); ada.pesapane@unicampania.it (A.P.); rosariaanna.fontanella@unicampania.it (R.A.F.); nunzia.balzano@unicampania.it (N.B.); nicoletta.lettera@unicampania.it (N.L.); albertamariamaddalena.palazzo@unicampania.it (A.M.M.P.); giovanni.tortorella@studenti.unicampania.it (G.T.); rashmi.joshi@unicampania.it (R.J.); asad.zia@unicampania.it (A.Z.); maryam.arshad@unicampania.it (M.A.); giuseppe.paolisso@unicampania.it (G.P.); 2 Unit of Internal Medicine III, Department of Medicine (DIMED), Padua University Hospital, University of Padua, 35128 Padua, Italy; federico.capone@unipd.it (F.C.); elisabetta.trevellin@unipd.it (E.T.); 3 Department of Biomedical Sciences, University of Padova, 35122 Padua, Italy; paola.fioretto@unipd.it; 4 Cardiology Unit, Sant’Andrea University Hospital, 00189 Rome, Italy; pasquale.paolisso@gmail.com (P.P.); belmontemarta1@gmail.com (M.B.); 5 Department of Clinical and Molecular Medicine, Sapienza University of Rome, 00185 Rome, Italy; 6 UniCamillus International University of Health Sciences, 00161 Rome, Italy

**Keywords:** ceramides, sphingolipid metabolism, cardiac metabolic dysfunction

## Abstract

**Background/Objectives:** Dietary patterns characterized by excess saturated fat intake contribute to obesity, type 2 diabetes, and cardiac metabolic dysfunction. Ceramides, bioactive sphingolipids synthesized in response to nutrient overload, have emerged as key molecular mediators linking dietary lipid composition to alterations in cardiac metabolic signaling. This review aims to integrate current evidence on diet-induced ceramide remodeling and its impact on intracellular pathways regulating cardiac metabolism. **Methods:** We analyzed experimental and clinical studies investigating the effects of high-fat and Western-type diets on myocardial ceramide synthesis, lipidomic remodeling, and downstream signaling pathways. Evidence from animal models, genetic and pharmacological interventions, nutritional studies, and circulating biomarker analyses was examined to delineate mechanistic and translational insights. **Results:** Saturated fatty acid excess, particularly palmitate, activates the de novo ceramide synthesis pathway in the myocardium, promoting accumulation of specific ceramide species. This remodeling impairs insulin signaling through Akt inhibition, protein phosphatase 2A activation, and PKCζ-dependent mechanisms, contributing to cardiac metabolic inflexibility. Ceramides further disrupt mitochondrial function by altering electron transport chain activity, increasing reactive oxygen species production, and modulating mitophagy and apoptotic signaling. Lipidomic studies highlight species-specific effects, with C16-ceramides frequently associated with adverse metabolic and cardiovascular outcomes, whereas very-long-chain ceramides may exert distinct functional roles. Circulating ceramide profiles have also been linked to diet-associated cardiovascular risk. **Conclusions:** Diet-induced ceramide remodeling represents a central molecular axis connecting dietary lipid excess to altered cardiac metabolic signaling. Targeting sphingolipid metabolism through nutritional or pharmacological strategies may offer novel opportunities for preventing and managing diet-associated cardiac dysfunction.

## 1. Introduction

The global rise in obesity and type 2 diabetes represents a major public health challenge and is closely associated with an increased risk of cardiovascular disease [1]. Among the organs affected by metabolic disturbances, the heart is particularly sensitive to alterations in nutrient availability and substrate utilization [2]. Under physiological conditions, the adult myocardium exhibits remarkable metabolic flexibility, being able to switch between fatty acids, glucose, lactate, and ketone bodies to meet its high energetic demands [3]. However, chronic nutrient excess—especially diets rich in saturated fatty acids and refined carbohydrates—can disrupt this metabolic adaptability, promoting lipid accumulation, mitochondrial dysfunction, and impaired cardiac performance [3]. In recent years, increasing attention has been directed toward the role of lipid intermediates in mediating the detrimental effects of nutrient overload on cardiac metabolism [4]. While triglyceride accumulation was initially considered the main hallmark of cardiac lipotoxicity, it is now widely recognized that bioactive lipid species, rather than neutral lipid storage, are major drivers of metabolic dysfunction [5]. Among these molecules, ceramides have emerged as key mediators linking dietary lipid excess to cellular stress responses and altered metabolic signaling [6]. Ceramides are sphingolipids generated through multiple pathways, including de novo synthesis from saturated fatty acids, sphingomyelin hydrolysis, and salvage pathways [7]. Notably, saturated fatty acid overload, including increased palmitate availability, promotes endogenous ceramide accumulation through the de novo sphingolipid pathway and contributes to insulin resistance and cardiac lipotoxicity [8,9,10]. Palmitate is a saturated fatty acid commonly enriched in Western-style diets [9,11]. Growing evidence indicates that ceramide accumulation plays a central role in the development of metabolic disorders, including insulin resistance, obesity, and cardiovascular disease [11]. In the heart, ceramides have been shown to interfere with insulin signaling pathways, impair mitochondrial function, and promote oxidative stress, inflammation, and apoptosis [12]. These effects ultimately contribute to cardiac metabolic inflexibility and structural remodeling, processes that are frequently observed in conditions such as obesity-related cardiomyopathy and diabetic cardiomyopathy [13]. Importantly, advances in lipidomic technologies have revealed that ceramides are not a homogeneous class of molecules. Distinct ceramide species, characterized by differences in acyl chain length and saturation, may exert diverse biological effects [7]. For instance, human lipidomic studies have shown that individual ceramide species and ceramide-based ratios are differentially associated with cardiometabolic risk. In the PREDIMED trial, plasma ceramide profiles were associated with cardiovascular disease, while adherence to a Mediterranean dietary pattern appeared to attenuate ceramide-associated cardiovascular risk [14]. CerS6-derived C16-ceramides have been shown in genetic mouse models to promote weight gain, glucose intolerance, and adverse metabolic remodeling, whereas very-long-chain ceramides may exert distinct and context-dependent biological functions [15,16]. In parallel, circulating ceramide profiles have recently gained attention as potential biomarkers of cardiometabolic risk, highlighting the translational relevance of sphingolipid metabolism in cardiovascular disease [17]. Moreover, emerging studies suggest that dietary composition and nutritional interventions may influence ceramide synthesis and distribution, providing new opportunities to modulate these pathways through lifestyle-based strategies [18]. Given the growing body of evidence linking diet, ceramide metabolism, and cardiac dysfunction, a comprehensive understanding of the molecular mechanisms involved is essential [19]. This review summarizes current knowledge on diet-induced ceramide remodeling in the heart, focusing on the molecular signaling pathways through which ceramides influence cardiac metabolism and function. In addition, we discuss species-specific effects of ceramides, their potential role as biomarkers of cardiovascular risk, and emerging nutritional and pharmacological approaches targeting sphingolipid metabolism.

## 2. Materials and Methods

### 2.1. Literature Search Strategy

This review was designed as a narrative review supported by a structured literature search, rather than as a systematic review or meta-analysis. Studies were selected narratively based on their relevance to the molecular mechanisms linking diet-induced ceramide remodeling and cardiac metabolic signaling, rather than through a formal systematic review screening process. A comprehensive literature search was conducted to identify studies investigating the relationship between dietary factors, ceramide metabolism, and cardiac metabolic dysfunction. Electronic databases including PubMed, Scopus, and Web of Science were searched for articles published up to March 2026. The search strategy combined relevant keywords and Medical Subject Headings (MeSH), including: ceramides, sphingolipids, lipidomics, cardiac metabolism, cardiomyocytes, lipotoxicity, diet, high-fat diet, saturated fatty acids, palmitate, cardiovascular disease, biomarkers, insulin resistance, and diabetic cardiomyopathy. Boolean operators (AND, OR) were used to refine the search and improve specificity. To ensure broad coverage of the literature, the reference lists of selected articles and recent review papers were manually screened to identify additional relevant studies.

### 2.2. Study Selection Criteria

Studies were selected based on their relevance to the molecular mechanisms linking diet-induced ceramide remodeling and cardiac metabolic signaling. The inclusion criteria were as follows: (i) experimental studies investigating ceramide metabolism in cardiac cells or myocardial tissue; (ii) animal studies evaluating the effects of dietary interventions, including high-fat diets and lipid overload, on ceramide accumulation and cardiac metabolism; (iii) clinical studies assessing circulating ceramides as biomarkers of cardiovascular or metabolic disease; (iv) review articles providing mechanistic or translational insights into sphingolipid metabolism in cardiometabolic disorders. Studies were excluded if they: (i) did not address ceramide metabolism or sphingolipid pathways; (ii) focused exclusively on non-cardiac tissues without clear cardiometabolic relevance; (iii) were not peer-reviewed (e.g., conference abstracts, editorials); (iv) were not available in English.

### 2.3. Data Extraction and Synthesis

Relevant information from the selected studies was qualitatively extracted, including study design, experimental model, type of dietary exposure, ceramide biosynthetic pathways, individual ceramide species investigated, lipidomic profiles, molecular signaling mechanisms, circulating biomarkers, and principal cardiometabolic outcomes. The evidence was synthesized narratively to provide an integrated and mechanistic overview of the role of ceramides in diet-associated cardiac metabolic dysfunction, with particular emphasis on insulin signaling pathways, mitochondrial function, oxidative stress, inflammatory responses, species-specific ceramide effects, and translational applications in cardiovascular risk assessment. To improve mechanistic interpretation, we distinguish, whenever possible, between studies investigating endogenous ceramide accumulation and those based on exogenous ceramide administration. Endogenous ceramides refer to ceramide species generated within cells or tissues through de novo synthesis, sphingomyelin hydrolysis, or salvage pathways, typically in response to nutrient excess, saturated fatty acid exposure, high-fat diet, or metabolic stress. In contrast, exogenous ceramides are experimentally administered to cultured cells or tissues to dissect specific downstream signaling pathways. This distinction is relevant because endogenous ceramide accumulation more closely reflects diet- or disease-associated sphingolipid remodeling, whereas exogenous ceramide exposure represents a reductionist experimental approach useful for identifying direct molecular targets and signaling mechanisms. Therefore, throughout the revised manuscript, evidence has been interpreted according to the experimental model used, including in vitro, in vivo, and clinical studies.

### 2.4. Methodological Considerations

Given the narrative nature of this review, the included studies were heterogeneous in terms of experimental models, dietary interventions, and analytical approaches. Both preclinical and clinical evidence were considered to provide a comprehensive overview of the current knowledge. While this approach allows integration of mechanistic and translational insights, it may also introduce variability in study design and outcome interpretation. As this review was conceived as a narrative synthesis rather than a systematic review or meta-analysis, we did not include a PRISMA flow diagram, perform a formal risk-of-bias assessment or standardized study quality appraisal, or conduct a quantitative meta-analysis. To facilitate the interpretation of the evidence discussed throughout this review, the principal mechanisms by which ceramides contribute to cardiac metabolic dysfunction, their downstream metabolic effects, and representative supporting evidence are summarized in Table 1. In addition, representative experimental, preclinical, and clinical studies supporting the mechanistic and translational conclusions of this review are summarized in Table 2.

## 3. Dietary Regulation of Ceramide Synthesis

Dietary patterns characterized by excessive caloric intake and high levels of saturated fatty acids are major contributors to cardiometabolic disorders [25]. In particular, Western-style diets, which are enriched in saturated fats and refined carbohydrates, promote systemic lipid overload and metabolic imbalance [26]. Under these conditions, circulating free fatty acids—especially palmitate—are increased and readily taken up by peripheral tissues, including the myocardium [27]. The heart relies heavily on fatty acid oxidation for energy production; however, chronic lipid oversupply exceeds the oxidative capacity of cardiomyocytes, leading to intracellular lipid accumulation [28]. While part of this lipid excess is stored as triglycerides, an increasing body of evidence indicates that bioactive lipid intermediates, rather than neutral lipid pools, are primarily responsible for lipotoxic effects [29]. Among these intermediates, ceramides have emerged as key mediators linking dietary lipid excess to cardiac metabolic dysfunction [6].

### 3.1. Fatty Acid Composition and De Novo Ceramide Synthesis

Dietary fatty acid composition plays a critical role in modulating ceramide biosynthesis. Saturated fatty acids, particularly palmitate, serve as direct substrates for the de novo ceramide synthesis pathway, thereby promoting ceramide production in metabolically active tissues, including the heart [7,30]. Elevated palmitoyl-CoA availability enhances the activity of serine palmitoyltransferase (SPT) [30], the rate-limiting enzyme in this pathway, leading to increased formation of sphingoid bases and subsequent ceramide accumulation. In contrast, unsaturated fatty acids—such as monounsaturated and polyunsaturated fatty acids—appear to exert neutral or protective effects on ceramide metabolism, either by reducing substrate availability for ceramide synthesis or by promoting lipid partitioning toward less harmful storage forms [31]. These observations highlight the importance of dietary fat quality, rather than quantity alone, in regulating sphingolipid metabolism [32]. In addition to fatty acids, other dietary factors, including caloric excess and altered glucose metabolism, may indirectly influence ceramide synthesis by modulating substrate flux and enzymatic activity within lipid metabolic pathways [33].

### 3.2. Clinical Evidence Linking Diet-Induced Ceramide Accumulation to Cardiometabolic Risk 

Clinical studies further support the relationship between diet, ceramide metabolism, and cardiovascular risk. Elevated circulating ceramide levels have been consistently associated with obesity, insulin resistance, metabolic syndrome, and cardiovascular disease. In particular, large cohort studies have identified specific ceramide species as strong predictors of adverse cardiovascular outcomes, leading to the development of clinically relevant ceramide-based risk scores [22]. Seminal studies by Laaksonen et al. [21] demonstrated that circulating ceramides, particularly Cer(d18:1/16:0), Cer(d18:1/18:0), and Cer(d18:1/24:1), were independently associated with cardiovascular mortality in patients with stable coronary artery disease, even after adjustment for conventional lipid parameters such as LDL cholesterol. These findings contributed to the development of the CERT (Coronary Event Risk Test) score, which integrates specific ceramide species and their ratios to improve cardiovascular risk stratification. Subsequent validation studies confirmed that elevated ceramide-based scores predict major adverse cardiovascular events, heart failure progression, and all-cause mortality across different patient populations [23]. Additional human studies have shown that circulating ceramide concentrations correlate closely with markers of metabolic dysfunction. Individuals with obesity and type 2 diabetes exhibit significantly increased plasma ceramide levels, particularly long-chain species associated with insulin resistance and impaired metabolic flexibility [20]. Importantly, these alterations appear to precede overt cardiovascular manifestations, suggesting that ceramides may act not only as biomarkers of disease progression but also as early indicators of cardiometabolic dysfunction [34]. Although direct quantification of myocardial ceramide accumulation in humans remains limited due to the invasive nature of cardiac tissue sampling, circulating ceramide profiles are increasingly considered reliable surrogate markers of systemic sphingolipid dysregulation. Lipidomic analyses have further revealed that distinct ceramide species exert differential clinical significance [35]. For example, elevated levels of C16- and C18-ceramides are frequently associated with adverse cardiometabolic outcomes, whereas very-long-chain species may display neutral or context-dependent effects [6]. Emerging evidence also indicates that dietary interventions can modulate circulating ceramide levels in humans. Adherence to Mediterranean-style dietary patterns, caloric restriction, and diets enriched in unsaturated fatty acids have been associated with reduced circulating ceramides and improved metabolic profiles [24]. In contrast, high intake of saturated fatty acids promotes ceramide accumulation and exacerbates insulin resistance and inflammatory signaling [24]. These observations reinforce the concept that dietary composition plays a critical role in regulating sphingolipid metabolism and may directly influence cardiovascular risk through modulation of ceramide pathways.

## 4. Ceramide-Mediated Molecular Signaling in Cardiomyocytes

Beyond their structural role in membrane organization, ceramides are increasingly recognized as potent bioactive lipids capable of modulating multiple intracellular signaling pathways involved in cardiac metabolism and stress adaptation [36]. Under conditions of nutrient excess, ceramide accumulation acts as a molecular transducer linking dietary lipid overload to metabolic dysfunction in cardiomyocytes. In this context, ceramides function not merely as passive byproducts of altered lipid metabolism but as active signaling mediators that influence insulin sensitivity, mitochondrial homeostasis, inflammatory responses, and cell survival [35]. Importantly, the biological effects of ceramides appear to depend on several factors, including acyl-chain composition, intracellular localization, and the specific enzymatic pathways involved in their synthesis. This complexity highlights the multifaceted role of ceramide signaling in the progression of diet-associated cardiac dysfunction [37].

### 4.1. Species-Specific Effects of Ceramides

Although ceramides are commonly considered a single class of bioactive sphingolipids, increasing evidence indicates that their biological effects are highly species-specific [7]. Differences in acyl-chain length, degree of saturation, subcellular localization, and the ceramide synthase isoform involved in their generation may substantially influence their metabolic and signaling properties [38]. A major determinant of ceramide heterogeneity is the substrate specificity of ceramide synthase (CerS) isoforms. CerS enzymes display partially overlapping but preferential acyl-chain specificities: CerS1 mainly generates C18-ceramides, CerS2 preferentially produces very-long-chain ceramides such as C22- and C24-ceramides, CerS4 contributes to C18–C20 species, whereas CerS5 and CerS6 are major sources of C16-ceramides. This isoform specificity is mechanistically relevant because changes in CerS expression or activity may alter not only total ceramide abundance but also the relative balance among individual ceramide species with potentially distinct biological effects [39]. In cardiac and metabolically active tissues, this balance may influence several cellular processes. Long-chain ceramides, particularly C16- and C18-ceramides, have been repeatedly associated with impaired insulin signaling, mitochondrial dysfunction, oxidative stress, apoptosis, and adverse cardiometabolic outcomes [39]. By contrast, very-long-chain ceramides, such as C22- and C24-ceramides, may exert more complex and context-dependent effects, potentially contributing to membrane organization, lipid raft stability, and cellular homeostasis under specific conditions [7]. These species-specific effects may also depend on subcellular compartmentalization. Ceramides generated in the endoplasmic reticulum through the de novo pathway may influence ER stress, lipid metabolic flux, and intracellular trafficking, whereas ceramides accumulating in mitochondrial-associated membranes or mitochondrial compartments may directly affect mitochondrial permeability, respiratory function, reactive oxygen species production, and apoptotic signaling. Therefore, the biological impact of ceramide accumulation is likely determined not only by the total amount of ceramides but also by the specific species generated, the CerS isoforms involved, and the cellular compartment in which these lipids accumulate [40]. However, whether C16-ceramides and very-long-chain ceramides exert intrinsically distinct biological functions or instead reflect different metabolic pools remains incompletely resolved [38,41]. Current evidence suggests that their effects are highly context-dependent and may vary according to cell type, nutritional status, CerS isoform expression, subcellular localization, and disease stage [38,39,40,41]. Therefore, species-specific ceramide profiles should not be interpreted simply as isolated biomarkers, but rather as indicators of broader sphingolipid remodeling with potential functional consequences for cardiac metabolic signaling [39,40,41]. This species-specific complexity is particularly relevant in the cardiovascular setting, where circulating ceramide profiles and ceramide-based risk scores have shown prognostic value beyond conventional lipid parameters [40]. Therefore, distinguishing among individual ceramide species is essential for interpreting mechanistic studies, understanding diet-induced sphingolipid remodeling, and translating lipidomic findings into clinically meaningful biomarkers or therapeutic targets [41]. The principal biological effects and clinical relevance of the major ceramide species discussed in this review are summarized in Table 3.

### 4.2. Ceramide-Induced Impairment of Insulin Signaling

**Figure 1:**
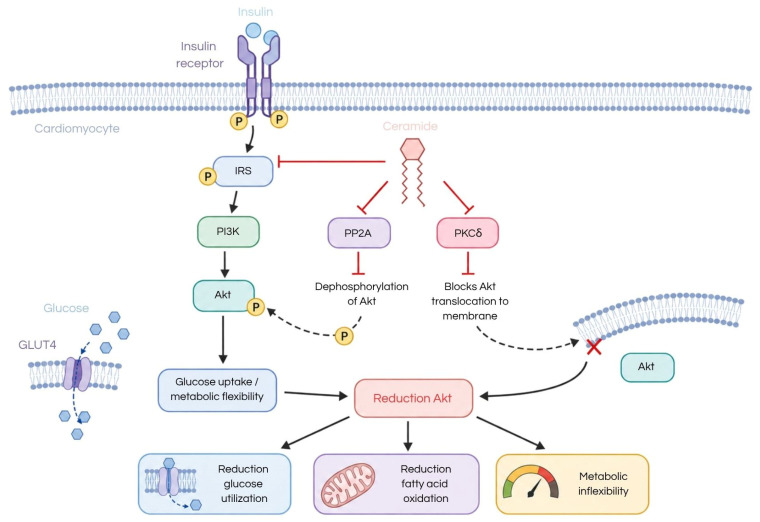
Ceramide-mediated impairment of insulin signaling in cardiomyocytes: Ceramide accumulation inhibits the IRS–PI3K–Akt pathway through PP2A-mediated Akt dephosphorylation and PKCζ-dependent blockade of Akt membrane translocation, leading to reduced glucose uptake, impaired substrate utilization, and metabolic inflexibility.

Evidence from both in vitro and in vivo studies has demonstrated that excessive ceramide accumulation disrupts insulin signaling pathways. In cultured cardiomyocytes and skeletal muscle cells, ceramides inhibit Akt activation through PP2A-mediated dephosphorylation and PKCζ-dependent impairment of Akt translocation to the plasma membrane [9,41]. In vivo, rodent models of high-fat diet-induced obesity have confirmed that increased endogenous ceramide synthesis contributes to myocardial insulin resistance and metabolic inflexibility [42]. Reduced Akt signaling impairs glucose utilization and shifts myocardial substrate preference toward excessive fatty acid oxidation, ultimately promoting metabolic inflexibility. In the context of chronic nutrient overload, this metabolic shift contributes to inefficient ATP production, increased oxygen consumption, and further lipid accumulation within cardiomyocytes [43]. Importantly, impaired insulin signaling is not only a metabolic abnormality but also a key contributor to the structural and functional remodeling observed in obesity-related and diabetic cardiomyopathy. Emerging evidence additionally suggests that ceramides may interfere with insulin receptor substrate (IRS) signaling and downstream nutrient-sensing pathways, further amplifying metabolic dysfunction under lipotoxic conditions (Figure 1) [43].

### 4.3. Mitochondrial Dysfunction and Oxidative Stress

Mitochondria are central regulators of cardiac energy metabolism, and their dysfunction represents a hallmark of diet-induced cardiometabolic disease. Ceramide accumulation has been strongly associated with mitochondrial abnormalities in cardiomyocytes, particularly under conditions of saturated fatty acid excess [44]. Several mechanisms have been proposed to explain ceramide-induced mitochondrial dysfunction. Ceramides can directly alter mitochondrial membrane permeability and disrupt electron transport chain (ETC) activity, leading to impaired oxidative phosphorylation and reduced ATP synthesis. In parallel, defective electron transport enhances the leakage of electrons and promotes excessive generation of reactive oxygen species (ROS) [45]. Increased oxidative stress contributes to lipid peroxidation, protein oxidation, mitochondrial DNA damage, and activation of stress-responsive signaling pathways. This creates a self-amplifying cycle in which mitochondrial dysfunction further enhances ceramide accumulation and lipotoxic signaling. Ceramides have also been implicated in the regulation of mitochondrial dynamics, including mitochondrial fission, fusion, and mitophagy [46]. Alterations in these processes impair mitochondrial quality control and promote the persistence of dysfunctional organelles within cardiomyocytes. Experimental studies indicate that specific ceramide species, particularly C16-ceramides, may preferentially localize to mitochondrial membranes and exert pronounced detrimental effects on mitochondrial integrity [47]. Given the exceptionally high energetic demands of the myocardium, even modest mitochondrial impairment may significantly compromise cardiac function over time [48].

### 4.4. Ceramides, Inflammation, and Cellular Stress Responses

In addition to metabolic dysregulation, ceramides contribute to the activation of inflammatory and stress-related signaling pathways in the heart. Excessive ceramide accumulation has been shown to activate nuclear factor κB (NF-κB), a major transcriptional regulator of inflammatory gene expression [7]. This activation promotes the production of pro-inflammatory cytokines and chemokines that contribute to myocardial inflammation and adverse cardiac remodeling. Ceramides have also been associated with activation of the NLRP3 inflammasome, a multiprotein complex involved in innate immune signaling and metabolic inflammation [49]. Inflammasome activation promotes maturation of interleukin-1β (IL-1β) and interleukin-18 (IL-18), thereby amplifying inflammatory responses within cardiac tissue. Simultaneously, ceramide accumulation induces endoplasmic reticulum (ER) stress through disruption of protein folding homeostasis and activation of the unfolded protein response (UPR) [50]. Persistent ER stress contributes to impaired cellular adaptation, calcium dysregulation, and apoptotic signaling. Importantly, these inflammatory and stress-related pathways do not occur independently but rather interact closely with mitochondrial dysfunction and insulin resistance, generating a complex network of maladaptive signaling events that accelerate cardiometabolic deterioration [51].

### 4.5. Regulation of Apoptosis and Autophagy

Ceramides are well-established regulators of cell fate and play a major role in apoptosis under conditions of metabolic stress. In cardiomyocytes, excessive ceramide accumulation promotes apoptotic signaling through both mitochondrial-dependent and receptor-mediated pathways [7]. At the mitochondrial level, ceramides facilitate outer mitochondrial membrane permeabilization, cytochrome c release, and activation of caspase cascades. These events ultimately result in programmed cell death and contribute to progressive loss of functional cardiomyocytes [52]. In parallel, ceramides influence autophagic pathways, although their effects appear to depend on the duration and severity of metabolic stress. Moderate activation of autophagy may initially represent a compensatory mechanism aimed at removing damaged organelles and maintaining cellular homeostasis [53]. However, chronic ceramide accumulation can dysregulate autophagic flux, leading to defective cellular quality control and further metabolic impairment. The balance between adaptive autophagy and apoptotic signaling is therefore likely to represent a critical determinant of cardiomyocyte survival during nutrient overload [54].

### 4.6. Integrated View of Ceramide Signaling in Diet-Associated Cardiac Dysfunction

Collectively, ceramides integrate multiple nutrient-sensitive signaling pathways involved in the pathogenesis of cardiac metabolic dysfunction [19]. Through simultaneous modulation of insulin signaling, mitochondrial homeostasis, oxidative stress, inflammation, ER stress, and cell survival pathways, ceramides act as central mediators of lipotoxic cardiac remodeling [55]. Importantly, these mechanisms are closely interconnected and may reinforce one another under conditions of chronic dietary lipid excess. This integrated signaling network ultimately contributes to the development of metabolic inflexibility, impaired cardiac energetics, and structural remodeling characteristics of obesity-related and diabetic heart disease [56]. The recognition of ceramides as active signaling mediators rather than passive lipid intermediates has important translational implications. Targeting ceramide metabolism and downstream signaling pathways may therefore represent a promising strategy to attenuate nutrient-induced cardiac dysfunction and improve cardiometabolic health [36].

## 5. Clinical and Translational Evidence

While experimental studies have provided substantial mechanistic insight into the role of ceramides in cardiac metabolic dysfunction, increasing efforts have been directed toward translating these findings into clinically relevant applications [15,16]. Evidence from both preclinical and human studies supports the concept that ceramide accumulation is closely associated with cardiometabolic disease progression and adverse cardiovascular outcomes [21]. In addition to their pathogenic role, circulating ceramides have emerged as promising biomarkers of cardiovascular risk and potential targets for nutritional and pharmacological interventions [23]. The following sections summarize the current preclinical and clinical evidence supporting the translational relevance of ceramide metabolism in cardiometabolic disease.

### 5.1. Preclinical Evidence

Preclinical studies provide strong mechanistic evidence supporting the role of ceramides as mediators of diet-induced cardiac metabolic dysfunction. In rodent models, chronic exposure to high-fat diets (HFDs) rich in saturated fatty acids induces myocardial ceramide accumulation, particularly through activation of the de novo sphingolipid synthesis pathway [8,57]. Increased availability of palmitoyl-CoA enhances serine palmitoyltransferase (SPT) activity, thereby promoting ceramide biosynthesis in cardiomyocytes [58]. Experimental evidence indicates that myocardial ceramide accumulation contributes directly to insulin resistance, mitochondrial dysfunction, oxidative stress, and apoptosis. Turpin et al. demonstrated that genetic modulation of ceramide synthesis significantly affects metabolic homeostasis, with ceramide accumulation promoting systemic and cardiac insulin resistance [15]. Similarly, Chaurasia et al. reported that CerS6-derived C16-ceramides contribute to mitochondrial fragmentation and impaired glucose metabolism in obesity-associated metabolic dysfunction [16]. In cardiac tissue, ceramide accumulation has also been linked to impaired mitochondrial oxidative phosphorylation and increased reactive oxygen species (ROS) production. Ussher and colleagues showed that lipid overload reduces metabolic flexibility in the myocardium and promotes maladaptive substrate utilization under obese and diabetic conditions [59]. Importantly, pharmacological inhibition of ceramide synthesis using agents such as myriocin attenuates lipotoxicity, improves insulin signaling, and partially restores cardiac metabolic function in experimental models [9]. Collectively, these findings strongly support a causal role of ceramides in nutrient-induced cardiac metabolic remodeling rather than a simple association with lipid overload.

### 5.2. Human Studies

Clinical studies further support the relationship between ceramide metabolism and cardiovascular disease. Human lipidomic studies have provided original clinical evidence supporting the role of circulating ceramides as biomarkers of cardiovascular and cardiometabolic risk. Elevated circulating ceramide levels have been consistently associated with obesity, insulin resistance, metabolic syndrome, coronary artery disease (CAD), and heart failure [20,21,22,23]. A landmark study by Laaksonen et al. demonstrated that specific plasma ceramides, particularly Cer(d18:1/16:0), Cer(d18:1/18:0), and Cer(d18:1/24:1), independently predicted cardiovascular death in patients with stable CAD and acute coronary syndromes beyond conventional lipid markers such as LDL cholesterol [21]. These findings represented a major advance in cardiovascular lipidomics and laid the foundation for the development of ceramide-based risk stratification tools. Subsequent studies confirmed that elevated circulating ceramide concentrations correlate with metabolic dysfunction and adverse cardiovascular outcomes. Haus et al. reported significantly increased plasma ceramide levels in obese individuals with type 2 diabetes, with strong associations between ceramides and insulin resistance indices [23]. Similarly, Poss et al. identified circulating sphingolipid signatures capable of predicting coronary artery disease independently of traditional cardiovascular risk factors [22]. Although direct quantification of myocardial ceramides in humans remains limited, circulating ceramide profiles are increasingly considered surrogate markers of systemic sphingolipid dysregulation and metabolic stress. Overall, these original clinical studies support the translational relevance of ceramide-based lipidomic profiling for cardiovascular risk stratification.

### 5.3. Ceramides as Cardiometabolic Biomarkers

One of the most clinically relevant aspects of ceramide biology is their emerging role as cardiovascular biomarkers. Advances in mass spectrometry-based lipidomics have enabled the identification of specific ceramide species strongly associated with cardiovascular risk and disease progression [60]. In particular, the CERT and CERT2 scores integrate circulating ceramide species and phospholipid ratios to improve cardiovascular risk prediction beyond traditional lipid parameters [61,62]. Elevated levels of Cer(d18:1/16:0), Cer(d18:1/18:0), and Cer(d18:1/24:1) have been associated with increased risk of myocardial infarction, heart failure, and cardiovascular mortality [21,61]. Importantly, ceramide-based risk scores appear to capture residual cardiovascular risk even in patients receiving optimal lipid-lowering therapies [62]. This suggests that sphingolipid metabolism may represent a pathogenic pathway distinct from classical cholesterol-mediated mechanisms. Recent evidence also suggests that distinct ceramide species exert differential biological and clinical effects. Long-chain ceramides, particularly C16 species, are frequently associated with insulin resistance and adverse cardiovascular outcomes, whereas very-long-chain ceramides may exert neutral or context-dependent effects [6,58]. These observations highlight the complexity of ceramide biology and underscore the importance of species-specific lipidomic profiling.

### 5.4. Analytical Approaches for Ceramide Quantification

The increasing clinical and translational interest in circulating ceramides has been largely driven by advances in mass spectrometry-based lipidomics. Ceramides are most commonly quantified in plasma, serum, or tissue extracts using liquid chromatography coupled to tandem mass spectrometry (LC-MS/MS), which provides high sensitivity and specificity for individual ceramide species [63]. Targeted lipidomics approaches, often performed on triple quadrupole instruments using multiple reaction monitoring, are particularly suitable for the accurate quantification of predefined ceramide species and are commonly used in studies evaluating ceramide-based cardiovascular risk scores such as CERT and CERT2 [21,61,64]. In contrast, untargeted or semi-targeted lipidomics platforms, frequently based on high-resolution mass spectrometry, allow broader profiling of sphingolipid remodeling and may identify additional lipid species or ratios associated with cardiometabolic risk [23,25,63].

Sample preparation is a critical step for reliable ceramide measurement. Lipids are typically extracted from biological samples using liquid–liquid extraction protocols, such as Folch- or Bligh and Dyer-based methods, or solid-phase extraction approaches when additional lipid class enrichment or sample cleanup is required [14,65]. Quantitative accuracy depends on the use of appropriate internal standards, including non-endogenous ceramide analogs such as C17-ceramide or stable isotope-labeled/deuterated ceramide standards, which correct for extraction efficiency, matrix effects, and analytical variability [63,64]. Analytical performance is commonly assessed through parameters such as lower limits of quantification, intra- and inter-assay coefficients of variation, linearity, recovery, and reproducibility across batches [63]. Several representative clinical lipidomic studies have applied these approaches to quantify circulating ceramides and derive clinically relevant risk scores. For example, Laaksonen et al. measured plasma ceramide species to demonstrate their ability to predict cardiovascular death beyond LDL cholesterol [21], while Hilvo et al. developed ceramide-phospholipid scores for residual cardiovascular risk prediction [61]. Similarly, Poss et al. used lipidomic and machine-learning approaches to identify serum sphingolipids as cholesterol-independent biomarkers of coronary artery disease [23], and Wang et al. applied plasma lipidomic profiling in the PREDIMED trial to evaluate the interaction between Mediterranean diet, ceramide profiles, and incident cardiovascular disease [24]. These examples highlight how analytical lipidomics has transformed ceramides from mechanistic lipid mediators into measurable biomarkers with potential clinical and nutritional relevance.

### 5.5. Dietary Interventions and Nutritional Modulation of Ceramide Metabolism

Emerging evidence indicates that dietary interventions can significantly modulate ceramide metabolism and circulating sphingolipid profiles. Nutritional strategies aimed at reducing saturated fatty acid intake and improving metabolic flexibility appear particularly relevant for limiting ceramide accumulation [24]. Adherence to Mediterranean dietary patterns has been associated with lower circulating ceramide concentrations and reduced cardiovascular risk, as observed in the PREDIMED trial, where higher adherence to a Mediterranean diet supplemented with extra-virgin olive oil or nuts was associated with favorable lipidomic profiles, including reduced levels of ceramide species linked to cardiovascular events [66]. Similarly, diets enriched in monounsaturated and polyunsaturated fatty acids may attenuate ceramide synthesis by reducing palmitate availability and promoting more favorable lipid partitioning [24]. Omega-3 fatty acids have also been reported to improve mitochondrial function and reduce inflammatory signaling pathways associated with ceramide accumulation [67]. Caloric restriction and exercise interventions further influence ceramide metabolism by improving insulin sensitivity and enhancing fatty acid oxidation [68]. Notably, reductions in circulating ceramide concentrations have been observed following weight loss interventions and lifestyle modification programs in individuals with obesity and metabolic syndrome [69]. Collectively, these findings support the concept that dietary composition directly influences sphingolipid metabolism and suggest that ceramide modulation may represent a mechanistic link between nutrition and cardiovascular health. Taken together, the available evidence supports a translational framework in which ceramides act at the interface between dietary exposures, metabolic dysfunction, and cardiovascular disease. Findings from preclinical models demonstrate a mechanistic role for ceramide accumulation in the development of insulin resistance, mitochondrial dysfunction, and lipotoxic cardiac remodeling, whereas human studies have identified specific ceramide species as robust predictors of adverse cardiometabolic outcomes. Importantly, the responsiveness of ceramide metabolism to dietary modification suggests that sphingolipid pathways may represent a biologically plausible mechanism through which nutritional interventions exert cardiovascular benefits. These observations strengthen the rationale for considering ceramides not only as biomarkers of cardiometabolic risk but also as potential targets for future preventive and therapeutic strategies.

## 6. Discussion

The evidence reviewed herein supports the concept that ceramides represent a critical molecular link between dietary nutrient excess and cardiac metabolic dysfunction. While alterations in substrate utilization and lipid accumulation are recognized features of obesity- and diabetes-associated cardiomyopathy, increasing evidence suggests that bioactive lipid intermediates, rather than neutral lipid storage itself, are major drivers of myocardial metabolic impairment [70]. In this context, ceramides emerge as central regulators of insulin sensitivity, mitochondrial homeostasis, inflammation, and cell survival [7]. A key observation from both experimental and clinical studies is that dietary lipid quality strongly influences ceramide accumulation. Saturated fatty acids, particularly palmitate, promote de novo ceramide synthesis, whereas dietary patterns rich in monounsaturated and polyunsaturated fatty acids, such as the Mediterranean diet, have been associated with lower circulating ceramide concentrations and improved cardiometabolic outcomes [36]. These findings support the hypothesis that some of the adverse cardiovascular effects of Western dietary patterns may be mediated through ceramide-dependent mechanisms. Recent advances in lipidomics have highlighted the biological heterogeneity of ceramides [10]. Long-chain species, particularly C16- and C18-ceramides, are consistently associated with insulin resistance, mitochondrial dysfunction, and adverse cardiovascular outcomes. However, not all ceramide species appear to exert uniformly detrimental effects. Very-long-chain ceramides may display more complex and context-dependent biological functions, emphasizing the need to move beyond measurements of total ceramide content and focus on species-specific effects and tissue distribution. The translational relevance of ceramide metabolism is further supported by the increasing use of circulating ceramides as biomarkers of cardiovascular risk [7]. Ceramide-based scores such as CERT and CERT2 have demonstrated prognostic value beyond conventional lipid parameters. Nevertheless, important challenges remain, including the lack of methodological standardization across lipidomic platforms and the need for validation in larger and more diverse populations before widespread clinical implementation [71]. Targeting ceramide metabolism represents a promising therapeutic strategy. Both pharmacological inhibition of ceramide synthesis and lifestyle interventions have shown beneficial effects on metabolic function and circulating ceramide levels. However, it remains unclear whether ceramides act primarily as causal mediators of disease progression or as sensitive biomarkers of broader metabolic disturbances [72]. Furthermore, much of the current mechanistic knowledge derives from animal models, and direct evidence from human myocardial tissue remains limited. Overall, diet-induced ceramide remodeling appears to play a central role in the pathogenesis of cardiac metabolic dysfunction [35]. However, the biological heterogeneity of ceramide species, the limited availability of human myocardial data, and the lack of standardized lipidomic methodologies highlight the need for further investigation [73]. A deeper understanding of the interactions between nutrition, sphingolipid metabolism, and cardiac signaling pathways may facilitate the development of precision nutrition approaches and novel therapeutic strategies aimed at reducing cardiometabolic risk [74].

### Limitations and Future Directions

Despite the growing evidence linking diet-induced ceramide remodeling to cardiac metabolic dysfunction, several aspects remain incompletely clarified. The current literature is characterized by substantial heterogeneity in experimental models, dietary interventions, biological matrices, lipidomic platforms, and analytical workflows. This heterogeneity may limit the direct comparison of findings across studies and should be considered when interpreting the translational relevance of ceramide profiles in cardiometabolic disease. A major unresolved issue concerns the relationship between circulating ceramides and tissue-specific sphingolipid remodeling. Although plasma and serum ceramide profiles have shown promising associations with cardiometabolic risk and cardiovascular outcomes, they may not fully reflect myocardial ceramide accumulation, subcellular compartmentalization, or local signaling events within cardiomyocytes. Direct evidence from human cardiac tissue remains limited, and further studies are needed to determine how circulating ceramide species relate to myocardial lipid remodeling, mitochondrial dysfunction, insulin signaling impairment, inflammation, and structural cardiac remodeling. Another important knowledge gap relates to the functional interpretation of individual ceramide species. Increasing evidence suggests that long-chain and very-long-chain ceramides may have distinct biological and clinical associations; however, whether these differences reflect intrinsically divergent biological functions, CerS isoform activity, subcellular localization, or broader metabolic remodeling remains to be fully established. Future studies should therefore integrate species-resolved lipidomics with mechanistic analyses of CerS isoform regulation, intracellular ceramide trafficking, and compartment-specific signaling pathways. From a nutritional and translational perspective, additional longitudinal and intervention studies are needed to clarify whether changes in ceramide profiles mediate the cardiovascular effects of dietary patterns, weight loss, exercise, or pharmacological modulation of sphingolipid metabolism. In particular, studies combining detailed dietary assessment, targeted and untargeted lipidomics, and functional cardiac phenotyping may help define whether ceramides act primarily as biomarkers of metabolic stress or as causal mediators of diet-associated cardiac dysfunction. Finally, improved standardization of lipidomic workflows will be essential for advancing the field. Harmonized protocols for sample collection, lipid extraction, internal standard selection, mass spectrometry acquisition, data processing, and reporting of analytical performance would increase reproducibility and facilitate comparison across studies. Such efforts may support the future implementation of ceramide-based biomarkers in precision nutrition, cardiovascular risk stratification, and targeted therapeutic strategies.

## 7. Conclusions

Diet-induced ceramide remodeling has emerged as a pivotal molecular mechanism linking nutritional excess to cardiac metabolic dysfunction. Current evidence indicates that excessive intake of saturated fatty acids promotes ceramide accumulation within the myocardium, triggering a complex network of maladaptive responses that includes impaired insulin signaling, mitochondrial dysfunction, oxidative stress, inflammation, and dysregulation of cell survival pathways. Together, these alterations contribute to metabolic inflexibility and progressive cardiac remodeling associated with obesity and type 2 diabetes. Advances in lipidomic technologies have further highlighted the biological heterogeneity of ceramide species, revealing distinct associations between specific ceramides and cardiometabolic risk. In parallel, the growing clinical utility of circulating ceramide profiles as biomarkers of cardiovascular disease underscores the translational relevance of sphingolipid metabolism. Importantly, accumulating evidence suggests that dietary interventions and lifestyle modifications can influence ceramide metabolism, supporting the concept that nutritional strategies may represent effective approaches to reduce ceramide burden and improve cardiometabolic health. Nevertheless, significant knowledge gaps remain regarding the tissue-specific functions of individual ceramide species, their causal contribution to cardiac dysfunction, and their responsiveness to long-term dietary interventions in humans. Overall, ceramides represent both promising biomarkers and potential therapeutic targets in diet-associated cardiovascular disease. A deeper understanding of the interactions between nutrition, sphingolipid metabolism, and cardiac signaling pathways may facilitate the development of precision nutrition and targeted therapeutic strategies aimed at preventing and mitigating obesity- and diabetes-related cardiac complications.

## Figures and Tables

**Table 1:** Main mechanisms linking diet-induced ceramide accumulation to cardiac metabolic dysfunction, associated metabolic consequences, and representative evidence.

Mechanism	Main Molecular Events	Consequences for Cardiac Metabolism	Representative Evidence
Increased ceramide synthesis	Saturated fatty acids (especially palmitate) activate de novo ceramide synthesis through SPT	Myocardial ceramide accumulation and lipotoxicity	High-fat diet and Western diet models
Impaired insulin signaling	Activation of PP2A and PKCζ inhibits Akt signaling	Reduced glucose utilization and metabolic inflexibility	Experimental studies in cardiomyocytes and animal models
Mitochondrial dysfunction	Altered ETC activity, reduced oxidative phosphorylation, increased ROS production	Impaired ATP generation and energetic inefficiency	Preclinical studies of lipid overload
Inflammation and ER stress	Activation of NF-κB, NLRP3 inflammasome and UPR pathways	Chronic inflammatory signaling and adverse remodeling	Experimental and translational studies
Apoptosis and autophagy dysregulation	Caspase activation and altered autophagic flux	Cardiomyocyte loss and impaired cellular homeostasis	Mechanistic studies
Ceramide species-specific effects	Differential actions of C16-, C18-, and very-long-chain ceramides	Variable cardiometabolic impact	Lipidomic studies
Clinical relevance	Increased circulating ceramides and elevated CERT/CERT2 scores	Higher risk of CAD, heart failure, and cardiovascular mortality	Human cohort studies
Nutritional modulation	Mediterranean diet, caloric restriction, and unsaturated fatty acids reduce ceramide burden	Improved cardiometabolic profile	Dietary intervention studies

**Table 2:** Representative experimental, preclinical, and clinical studies linking diet-induced ceramide remodeling to cardiometabolic dysfunction.

Author/Year	Study Type	Experimental model/Population	Dietary or Metabolic Exposure/Intervention	Ceramide Species/Pathway Investigated	Analytical Platform/Approach	Key Findings
Holland et al., 2007 [8]	Preclinical/mechanistic	Rodent models of saturated fat-, glucocorticoid-, and obesity-induced insulin resistance	Saturated fat exposure, obesity, glucocorticoid treatment; inhibition of ceramide synthesis	De novo ceramide synthesis pathway	Biochemical and metabolic analyses	Inhibition of ceramide synthesis ameliorated saturated fat- and obesity-induced insulin resistance.
Park et al., 2008 [9]	Preclinical/cardiac	Experimental model of lipotoxic cardiomyopathy	Cardiac lipid overload	Cardiac ceramide accumulation	Lipid and metabolic analyses	Ceramide accumulation contributed to lipotoxic cardiomyopathy and cardiac dysfunction.
Butler et al., 2017 [10]	Preclinical/nutritional	Healthy and hypertrophied hearts	Western diet exposure	Cardiac ceramide content	Cardiac lipid analysis	Western diet increased cardiac ceramide content in healthy and hypertrophied hearts.
Turpin et al., 2014 [15]	Preclinical/genetic	Mouse models of obesity-associated metabolic dysfunction	Obesity-related metabolic stress; CerS6 modulation	CerS6-derived C16:0 ceramide	Genetic, lipidomic, and metabolic analyses	CerS6-dependent C16:0 ceramide production promoted weight gain and glucose intolerance.
Chaurasia et al., 2019 [16]	Preclinical/mechanistic	Experimental models of obesity-associated insulin resistance	Targeting ceramide desaturation/remodeling	Ceramide remodeling and ceramide double bond	Genetic/pharmacological and metabolic analyses	Targeting ceramide remodeling improved insulin resistance and hepatic steatosis.
Haus et al., 2009 [20]	Human clinical	Obese subjects with type 2 diabetes	Obesity and type 2 diabetes	Plasma ceramides	Plasma lipid analysis	Plasma ceramides were elevated and correlated with the severity of insulin resistance.
Laaksonen et al., 2016 [21]	Human clinical	Patients with stable coronary artery disease and acute coronary syndromes	Cardiovascular disease cohorts	Plasma ceramides, including Cer(d18:1/16:0), Cer(d18:1/18:0), and Cer(d18:1/24:1)	Plasma lipidomics	Plasma ceramides predicted cardiovascular death beyond conventional lipid markers, including LDL cholesterol.
Hilvo et al., 2020 [22]	Human clinical	Patients with stable coronary heart disease receiving optimal medical therapy	Residual cardiovascular risk under optimal therapy	Ceramide-phospholipid score	Plasma lipidomics and risk-score analysis	Ceramide-phospholipid score improved prediction of residual cardiovascular risk.
Poss et al., 2020 [23]	Human clinical/lipidomics	Human cohort for coronary artery disease assessment	Coronary artery disease (CAD)	Serum sphingolipids and ceramide-related lipid signatures	Machine learning and serum lipidomics	Serum sphingolipids were identified as cholesterol-independent biomarkers of coronary artery disease.
Wang et al., 2017 [24]	Human nutritional cohort	PREDIMED trial population	Mediterranean diet intervention supplemented with extra-virgin olive oil or nuts	Plasma ceramides	Plasma lipidomics	Plasma ceramide profiles were associated with incident cardiovascular disease, and the Mediterranean diet appeared to attenuate ceramide-associated cardiovascular risk.

Abbreviations: CAD, coronary artery disease; CerS6, ceramide synthase 6; LDL, low-density lipoprotein; PREDIMED, Prevención con Dieta Mediterránea.

**Table 3:** Major ceramide species, their biosynthetic enzymes, biological functions, and clinical relevance.

Ceramide Species	Main Source/Enzyme	Principal Biological Effects	Clinical Relevance
**Cer(d18:1/16:0)** (C16-ceramide)	Mainly CerS6	Insulin resistance, mitochondrial dysfunction, ROS production, apoptosis	Strongly associated with obesity, T2D, CAD, and cardiovascular mortality
**Cer(d18:1/18:0)** (C18-ceramide)	CerS1	Impaired glucose metabolism, metabolic inflexibility, cellular stress responses	Associated with cardiometabolic dysfunction and adverse cardiovascular outcomes
**Cer(d18:1/20:0)**	CerS4	Regulation of membrane organization and cellular signaling	Emerging biomarker in cardiometabolic disease
**Cer(d18:1/22:0)**	CerS2	Modulation of lipid homeostasis and membrane stability	Variable association with cardiovascular risk
**Cer(d18:1/24:0)** (Very-long-chain ceramide)	CerS2	Maintenance of membrane integrity and cellular homeostasis	May exert neutral or potentially protective effects in some settings
**Cer(d18:1/24:1)**	CerS2	Regulation of lipid metabolism and cellular stress responses	Included in CERT/CERT2 scores and associated with cardiovascular risk prediction

CerS, ceramide synthase; ROS, reactive oxygen species; T2D, type 2 diabetes; CAD, coronary artery disease; CERT, Coronary Event Risk Test.

## Data Availability

The original contributions presented in this study are included in the article. Further inquiries can be directed to the corresponding author.

## Abbreviations

The following abbreviations are used in this manuscript:

Akt	Protein Kinase B
ATP	Adenosine Triphosphate
CAD	Coronary Artery Disease
CERT	Coronary Event Risk Test
CerS6	Ceramide Synthase 6
ER	Endoplasmic Reticulum
ETC	Electron Transport Chain
HFD	High-Fat Diet
IL-1β	Interleukin-1 Beta
IL-18	Interleukin-18
IRS	Insulin Receptor Substrate
LDL	Low-Density Lipoprotein
NF-κB	Nuclear Factor Kappa B
NLRP3	NOD-, LRR- and Pyrin Domain-Containing Protein 3
PI3K	Phosphoinositide 3-Kinase
PKCζ	Protein Kinase C Zeta
PP2A	Protein Phosphatase 2A
ROS	Reactive Oxygen Species
SPT	Serine Palmitoyltransferase

## References

[1] Scherer P.E., Hill J.A. (2016). Obesity, Diabetes, and Cardiovascular Diseases: A Compendium. Circ. Res..

[2] Xia J., Nong Y., Teng J., Mohammed S.A., Liu J., Pang Y., Costantino S., Ruschitzka F., Hamdani N., Abdellatif M. (2025). Unlocking metabolic flexibility in heart failure with preserved ejection fraction: Bridging fundamental mechanisms to clinical innovation. iScience.

[3] Guan X., Zhou C., Zhuo X., Yang W., Wang H. (2025). The heart of diabetes: Unraveling metabolic drivers of cardiomyopathy. Cardiovasc. Diabetol. Endocrinol. Rep..

[4] Li C., Wang Z., Yang Y., Jiang Q., Jiang Y., Xiao J., Shen L., Wu W., Li C. (2026). Pro-resolving lipid mediators in diseases: Exploring the molecular basis and clinical implication. Mol. Biomed..

[5] Lair B., Laurens C., Van Den Bosch B., Moro C. (2020). Novel Insights and Mechanisms of Lipotoxicity-Driven Insulin Resistance. Int. J. Mol. Sci..

[6] Chaurasia B., Summers S.A. (2021). Ceramides in Metabolism: Key Lipotoxic Players. Annu. Rev. Physiol..

[7] Gonzalez-Plascencia M., Garza-Veloz I., Flores-Morales V., Martinez-Fierro M.L. (2026). The Role of Ceramides in Metabolic and Cardiovascular Diseases. J. Cardiovasc. Dev. Dis..

[8] Holland W.L., Brozinick J.T., Wang L.P., Hawkins E.D., Sargent K.M., Liu Y., Narra K., Hoehn K.L., Knotts T.A., Siesky A. (2007). Inhibition of ceramide synthesis ameliorates glucocorticoid-, saturated-fat-, and obesity-induced insulin resistance. Cell Metab..

[9] Park T.S., Hu Y., Noh H.L., Drosatos K., Okajima K., Buchanan J., Tuinei J., Homma S., Jiang X.C., Abel E.D. (2008). Ceramide is a cardiotoxin in lipotoxic cardiomyopathy. J. Lipid Res..

[10] Butler T.J., Ashford D., Seymour A.M. (2017). Western diet increases cardiac ceramide content in healthy and hypertrophied hearts. Nutr. Metab. Cardiovasc. Dis..

[11] Annevelink C.E., Sapp P.A., Petersen K.S., Shearer G.C., Kris-Etherton P.M. (2023). Diet-derived and diet-related endogenously produced palmitic acid: Effects on metabolic regulation and cardiovascular disease risk. J. Clin. Lipidol..

[12] Yang S., Wu Y. (2025). Ceramide metabolism and cardiovascular risk factors: Insights into therapeutic strategies. Front. Cardiovasc. Med..

[13] Fukushima A., Lopaschuk G.D. (2016). Cardiac fatty acid oxidation in heart failure associated with obesity and diabetes. Biochim. Biophys. Acta.

[14] Folch J., Lees M., Sloane Stanley G.H. (1957). A simple method for the isolation and purification of total lipides from animal tissues. J. Biol. Chem..

[15] Turpin S.M., Nicholls H.T., Willmes D.M., Mourier A., Brodesser S., Wunderlich C.M., Mauer J., Xu E., Hammerschmidt P., Bronneke H.S. (2014). Obesity-induced CerS6-dependent C16:0 ceramide production promotes weight gain and glucose intolerance. Cell Metab..

[16] Chaurasia B., Tippetts T.S., Mayoral Monibas R., Liu J., Li Y., Wang L., Wilkerson J.L., Sweeney C.R., Pereira R.F., Sumida D.H. (2019). Targeting a ceramide double bond improves insulin resistance and hepatic steatosis. Science.

[17] Petrocelli J.J., McKenzie A.I., Mahmassani Z.S., Reidy P.T., Stoddard G.J., Poss A.M., Holland W.L., Summers S.A., Drummond M.J. (2020). Ceramide Biomarkers Predictive of Cardiovascular Disease Risk Increase in Healthy Older Adults After Bed Rest. J. Gerontol. A Biol. Sci. Med. Sci..

[18] Wang S., Jin Z., Wu B., Morris A.J., Deng P. (2025). Role of dietary and nutritional interventions in ceramide-associated diseases. J. Lipid Res..

[19] Shalaby Y.M., Al Aidaros A., Valappil A., Ali B.R., Akawi N. (2021). Role of Ceramides in the Molecular Pathogenesis and Potential Therapeutic Strategies of Cardiometabolic Diseases: What we Know so Far. Front. Cell Dev. Biol..

[20] Haus J.M., Kashyap S.R., Kasumov T., Zhang R., Kelly K.R., Defronzo R.A., Kirwan J.P. (2009). Plasma ceramides are elevated in obese subjects with type 2 diabetes and correlate with the severity of insulin resistance. Diabetes.

[21] Laaksonen R., Ekroos K., Sysi-Aho M., Hilvo M., Vihervaara T., Kauhanen D., Suoniemi M., Hurme R., Marz W., Scharnagl H. (2016). Plasma ceramides predict cardiovascular death in patients with stable coronary artery disease and acute coronary syndromes beyond LDL-cholesterol. Eur. Heart J..

[22] Hilvo M., Wallentin L., Ghukasyan Lakic T., Held C., Kauhanen D., Jylha A., Lindback J., Siegbahn A., Granger C.B., Koenig W. (2020). Prediction of Residual Risk by Ceramide-Phospholipid Score in Patients With Stable Coronary Heart Disease on Optimal Medical Therapy. J. Am. Heart Assoc..

[23] Poss A.M., Maschek J.A., Cox J.E., Hauner B.J., Hopkins P.N., Hunt S.C., Holland W.L., Summers S.A., Playdon M.C. (2020). Machine learning reveals serum sphingolipids as cholesterol-independent biomarkers of coronary artery disease. J. Clin. Investig..

[24] Wang D.D., Toledo E., Hruby A., Rosner B.A., Willett W.C., Sun Q., Razquin C., Zheng Y., Ruiz-Canela M., Guasch-Ferre M. (2017). Plasma Ceramides, Mediterranean Diet, and Incident Cardiovascular Disease in the PREDIMED Trial (Prevencion con Dieta Mediterranea). Circulation.

[25] Wang W., Liu Y., Li Y., Luo B., Lin Z., Chen K., Liu Y. (2023). Dietary patterns and cardiometabolic health: Clinical evidence and mechanism. MedComm.

[26] Clemente-Suarez V.J., Beltran-Velasco A.I., Redondo-Florez L., Martin-Rodriguez A., Tornero-Aguilera J.F. (2023). Global Impacts of Western Diet and Its Effects on Metabolism and Health: A Narrative Review. Nutrients.

[27] Wanders R.J.A., Visser G., Ferdinandusse S., Vaz F.M., Houtkooper R.H. (2020). Mitochondrial Fatty Acid Oxidation Disorders: Laboratory Diagnosis, Pathogenesis, and the Complicated Route to Treatment. J. Lipid Atheroscler..

[28] Yan A., Xie G., Ding X., Wang Y., Guo L. (2021). Effects of Lipid Overload on Heart in Metabolic Diseases. Horm. Metab. Res..

[29] Sethi P., Mishra A.K., Ghosh S., Singh K.K., Sharma S., Stojchevski R., Avtanski D., Sinha J.K. (2025). Lipid Metabolism-Signaling Crosstalk in Metabolic Disease and Aging: Mechanisms and Therapeutic Targets. Nutrients.

[30] Imierska M., Zabielski P., Roszczyc-Owsiejczuk K., Sokolowska E., Pogodzinska K., Kojta I., Blachnio-Zabielska A. (2022). Serine Palmitoyltransferase Gene Silencing Prevents Ceramide Accumulation and Insulin Resistance in Muscles in Mice Fed a High-Fat Diet. Cells.

[31] Pralhada Rao R., Vaidyanathan N., Rengasamy M., Mammen Oommen A., Somaiya N., Jagannath M.R. (2013). Sphingolipid metabolic pathway: An overview of major roles played in human diseases. J. Lipids.

[32] Voss J., Hornemann T., Belgardt B.F. (2025). Impact of Nutrition on Sphingolipid-Regulated Physiology: A Review. Mol. Nutr. Food Res..

[33] Reginato A., Veras A.C.C., Baqueiro M.D.N., Panzarin C., Siqueira B.P., Milanski M., Lisboa P.C., Torsoni A.S. (2021). The Role of Fatty Acids in Ceramide Pathways and Their Influence on Hypothalamic Regulation of Energy Balance: A Systematic Review. Int. J. Mol. Sci..

[34] Gaggini M., Vassalle C. (2026). Ceramides as Biomarkers and Pharmacological Targets in Heart Failure Pathophysiology. Biomolecules.

[35] Chen X., Fonseka O., Han Y., Liu W. (2026). Ceramides in the Heart: Physiological and Pathological Roles and Regulation. Cells.

[36] Arias-Marroquin A.T., Torre-Villalvazo I., Granados Portillo O., Villegas-Romero M., Camacho-Morales A., Tovar A.R., Aguilar Salinas C.A., Illescas-Zarate D., Vazquez-Manjarrez N. (2026). Modulation of ceramides through nutrition: A new target in obesity and insulin resistance (Narrative Review). Clin. Nutr. ESPEN.

[37] Thakkar H., Vincent V., Chaurasia B. (2025). Ceramide signaling in immunity: A molecular perspective. Lipids Health Dis..

[38] Mullen T.D., Hannun Y.A., Obeid L.M. (2012). Ceramide synthases at the centre of sphingolipid metabolism and biology. Biochem. J..

[39] Zietzer A., Dusing P., Reese L., Nickenig G., Jansen F. (2022). Ceramide Metabolism in Cardiovascular Disease: A Network With High Therapeutic Potential. Arterioscler. Thromb. Vasc. Biol..

[40] Klingenberg R., Leiherer A., Dobrev D., Kaski J.C., Levkau B., Marz W., Sossalla S., von Eckardstein A., Drexel H. (2025). Ceramides in cardiovascular disease: Emerging role as independent risk predictors and novel therapeutic targets. Cardiovasc. Res..

[41] Caturano A., Galiero R., Vetrano E., Sardu C., Rinaldi L., Russo V., Monda M., Marfella R., Sasso F.C. (2024). Insulin-Heart Axis: Bridging Physiology to Insulin Resistance. Int. J. Mol. Sci..

[42] Hage Hassan R., Pacheco de Sousa A.C., Mahfouz R., Hainault I., Blachnio-Zabielska A., Bourron O., Koskas F., Gorski J., Ferre P., Foufelle F. (2016). Sustained Action of Ceramide on the Insulin Signaling Pathway in Muscle Cells: Implication of the double-stranded rna-activated protein kinase. J. Biol. Chem..

[43] Smith R.L., Soeters M.R., Wust R.C.I., Houtkooper R.H. (2018). Metabolic Flexibility as an Adaptation to Energy Resources and Requirements in Health and Disease. Endocr. Rev..

[44] Werbner B., Tavakoli-Rouzbehani O.M., Fatahian A.N., Boudina S. (2023). The dynamic interplay between cardiac mitochondrial health and myocardial structural remodeling in metabolic heart disease, aging, and heart failure. J. Cardiovasc. Aging.

[45] McCaffrey J.M., Ibdah J.A. (2025). Effects of Diet and Exercise on Mitochondrial Health in Metabolic Dysfunction-Associated Steatotic Liver Disease (MASLD): Role of Ceramides. Nutrients.

[46] Liu S., Liu J., Wang Y., Deng F., Deng Z. (2025). Oxidative Stress: Signaling Pathways, Biological Functions, and Disease. MedComm.

[47] Park L.K., Garr Barry V., Hong J., Heebink J., Sah R., Peterson L.R. (2022). Links between ceramides and cardiac function. Curr. Opin. Lipidol..

[48] Paraskevaidis I., Kourek C., Farmakis D., Tsougos E. (2024). Mitochondrial Dysfunction in Cardiac Disease: The Fort Fell. Biomolecules.

[49] Suetomi T., Willeford A., Brand C.S., Cho Y., Ross R.S., Miyamoto S., Brown J.H. (2018). Inflammation and NLRP3 Inflammasome Activation Initiated in Response to Pressure Overload by Ca^2+^/Calmodulin-Dependent Protein Kinase II delta Signaling in Cardiomyocytes Are Essential for Adverse Cardiac Remodeling. Circulation.

[50] Liao Y., Liu K., Zhu L. (2022). Emerging Roles of Inflammasomes in Cardiovascular Diseases. Front. Immunol..

[51] Ghemrawi R., Battaglia-Hsu S.F., Arnold C. (2018). Endoplasmic Reticulum Stress in Metabolic Disorders. Cells.

[52] Hearps A.C., Burrows J., Connor C.E., Woods G.M., Lowenthal R.M., Ragg S.J. (2002). Mitochondrial cytochrome c release precedes transmembrane depolarisation and caspase-3 activation during ceramide-induced apoptosis of Jurkat T cells. Apoptosis.

[53] Jiang W., Ogretmen B. (2014). Autophagy paradox and ceramide. Biochim. Biophys. Acta.

[54] Yun H.R., Singh M.K., Han S., Ranbhise J.S., Ha J., Kim S.S., Kang I. (2025). Roles of Autophagy and Oxidative Stress in Cardiovascular Disease. Antioxidants.

[55] Galizzi G., Di Carlo M. (2022). Insulin and Its Key Role for Mitochondrial Function/Dysfunction and Quality Control: A Shared Link between Dysmetabolism and Neurodegeneration. Biology.

[56] Capasso L., Mele D., Casalino R., Favale G., Rollo G., Verrilli G., Conte M., Bontempo P., Carafa V., Altucci L. (2025). Nutritional Regulation of Cardiac Metabolism and Function: Molecular and Epigenetic Mechanisms and Their Role in Cardiovascular Disease Prevention. Nutrients.

[57] Bikman B.T., Summers S.A. (2011). Ceramides as modulators of cellular and whole-body metabolism. J. Clin. Investig..

[58] Wilkerson J.L., Tatum S.M., Holland W.L., Summers S.A. (2024). Ceramides are fuel gauges on the drive to cardiometabolic disease. Physiol. Rev..

[59] Actis Dato V., Lange S., Cho Y. (2024). Metabolic Flexibility of the Heart: The Role of Fatty Acid Metabolism in Health, Heart Failure, and Cardiometabolic Diseases. Int. J. Mol. Sci..

[60] Meikle P.J., Summers S.A. (2017). Sphingolipids and phospholipids in insulin resistance and related metabolic disorders. Nat. Rev. Endocrinol..

[61] Hilvo M., Simolin H., Metso J., Ruuth M., Oorni K., Jauhiainen M., Laaksonen R., Baruch A. (2018). PCSK9 inhibition alters the lipidome of plasma and lipoprotein fractions. Atherosclerosis.

[62] Poss A.M., Summers S.A. (2020). Too Much of a Good Thing? An Evolutionary Theory to Explain the Role of Ceramides in NAFLD. Front. Endocrinol..

[63] Bowden J.A., Heckert A., Ulmer C.Z., Jones C.M., Koelmel J.P., Abdullah L., Ahonen L., Alnouti Y., Armando A.M., Asara J.M. (2017). Harmonizing lipidomics: NIST interlaboratory comparison exercise for lipidomics using SRM 1950-Metabolites in Frozen Human Plasma. J. Lipid Res..

[64] Sullards M.C., Allegood J.C., Kelly S., Wang E., Haynes C.A., Park H., Chen Y., Merrill A.H. (2007). Structure-specific, quantitative methods for analysis of sphingolipids by liquid chromatography-tandem mass spectrometry: “inside-out” sphingolipidomics. Methods Enzymol..

[65] Bligh E.G., Dyer W.J. (1959). A rapid method of total lipid extraction and purification. Can. J. Biochem. Physiol..

[66] Razquin C., Ruiz-Canela M., Wernitz A., Toledo E., Corella D., Alonso-Gomez A., Fito M., Gomez-Gracia E., Estruch R., Fiol M. (2023). Effects of Supplemented Mediterranean Diets on Plasma-Phospholipid Fatty Acid Profiles and Risk of Cardiovascular Disease after 1 Year of Intervention in the PREDIMED Trial. Clin. Chem..

[67] Innes J.K., Calder P.C. (2020). Marine Omega-3 (N-3) Fatty Acids for Cardiovascular Health: An Update for 2020. Int. J. Mol. Sci..

[68] Bergman B.C., Brozinick J.T., Strauss A., Bacon S., Kerege A., Bui H.H., Sanders P., Siddall P., Wei T., Thomas M.K. (2016). Muscle sphingolipids during rest and exercise: A C18:0 signature for insulin resistance in humans. Diabetologia.

[69] Bonora E. (2000). Relationship between regional fat distribution and insulin resistance. Int. J. Obes. Relat. Metab. Disord..

[70] Han Y., Chen X., Fonseka O., Liu W. (2026). Lipotoxicity in Diabetic Cardiomyopathy: Molecular Basis and Emerging Therapeutic Targets. Int. J. Mol. Sci..

[71] Witoslawska A., Meessen J., Hilvo M., Jylha A., Zannad F., Cerrato M., Rossignol P., Novelli D., Duarte K., Targher G. (2025). Ceramide and phosphatidylcholine lipids-based risk score predicts major cardiovascular outcomes in patients with heart failure. Eur. J. Clin. Investig..

[72] Aburasayn H., Al Batran R., Ussher J.R. (2016). Targeting ceramide metabolism in obesity. Am. J. Physiol. Endocrinol. Metab..

[73] Shen X., Feng R., Zhou R., Zhang Z., Liu K., Wang S. (2025). Ceramide as a Promising Tool for Diagnosis and Treatment of Clinical Diseases: A Review of Recent Advances. Metabolites.

[74] Hamouche R., Summers S.A., Holland W.L., Navankasattusas S., Drakos S.G., Tseliou E. (2025). The role of sphingolipids in heart failure. Eur. Heart J. Open.

